# *Leishmania infantum HSP70-II* null mutant as candidate vaccine against leishmaniasis: a preliminary evaluation

**DOI:** 10.1186/1756-3305-4-150

**Published:** 2011-07-27

**Authors:** Javier Carrión, Cristina Folgueira, Manuel Soto, Manuel Fresno, Jose M Requena

**Affiliations:** 1 Centro de Biología Molecular "Severo Ochoa" (CSIC-UAM), Universidad Autónoma de Madrid, Madrid, Spain

## Abstract

**Background:**

Visceral leishmaniasis is the most severe form of leishmaniasis and no effective vaccine exists. The use of live attenuated vaccines is emerging as a promising vaccination strategy.

**Results:**

In this study, we tested the ability of a *Leishmania infantum *deletion mutant, lacking both *HSP70-II *alleles (ΔHSP70-II), to provide protection against *Leishmania *infection in the *L. major*-BALB/c infection model. Administration of the mutant line by either intraperitoneal, intravenous or subcutaneous route invariably leads to the production of high levels of NO and the development in mice of type 1 immune responses, as determined by analysis of anti-*Leishmania *IgG subclasses. In addition, we have shown that ΔHSP70-II would be a safe live vaccine as immunodeficient SCID mice, and hamsters (*Mesocricetus auratus*), infected with mutant parasites did not develop any sign of pathology.

**Conclusions:**

The results suggest that the ΔHSP70-II mutant is a promising and safe vaccine, but further studies in more appropriate animal models (hamsters and dogs) are needed to appraise whether this attenuate mutant would be useful as vaccine against visceral leishmaniasis.

## Background

Leishmaniasis is a vector-borne disease that is caused by the infection of protozoan parasites of the genus *Leishmania*. The extracellular promastigote forms of *Leishmania *are inoculated into humans (and other mammalian hosts) by sandflies (phlebotomine insects), after which the parasites undergo phagocytosis by macrophages and transform to intracellular amastigotes. Clinical manifestations of leishmaniasis are particularly diverse [1], ranging from subclinical (unapparent infections) to visceral leishmaniasis (VL), which is usually fatal when untreated. Other common forms of the disease are mucocutaneous (MCL), diffuse cutaneous (DCL) and cutaneous leishmaniasis (CL). The clinical outcomes depends upon a number of factors, including the species (and strain) of the parasite, as well as the host's genetically determined immune responses. Thus, *Leishmania major *and many other *Leishmania *species cause CL, *Leishmania donovani *and *Leishmania infantum *are mainly associated with VL, whereas MCL results after infection with parasites from the *Leishmania braziliensis *complex [2].

Leishmaniasis threatens 350 million people worldwide, mainly in developing countries. Annual incidence is estimated at 2 million cases and the overall prevalence is 12 million people [3]. In developing and under-developed parts of the world, AIDS and other immunosuppressive syndromes add to the higher risk of leishmaniasis [4]. In spite of its incidence, leishmaniasis is a neglected disease. Current control strategies rely on reservoir and vector control and pharmacological drugs, but new treatment strategies are clearly needed [5]. Abundant clinical and experimental evidence indicates that leishmaniasis would be preventable by vaccination, but anti-leishmanial vaccines for human use have yet to be developed [6-10].

Effective vaccination against human CL has been practiced for centuries by deliberate inoculation of living organisms from the exudates of active lesions or, more recently, by the inoculation of cultured *Leishmania *promastigotes (process known as "leishmanization") [11]. The appearance of complications, i.e. developing of severe disease in some individuals, led to abandoning the use of live *Leishmania *as a prophylactic vaccine. Nevertheless, leishmanization is still currently practiced in some countries including Uzbekistan, Afghanistan, Iraq, and Iran [12], and there are recent efforts to standardize it as a live vaccine and also to use it for rapidly assessing the efficacy of new vaccines [13]. On the other hand, first generation vaccines (or killed vaccines), prepared using inactivated whole parasites, have been the subject of many studies over decades and are the only vaccine candidates for leishmaniasis which have undergone phase 3 clinical trials. However, evidence of protective efficacy has not emerged from those clinical trials [14].

The ineffectiveness of vaccines based on either killed parasites or recombinant proteins seems to be a consequence of the short-term immunity they induce [15]. On the other hand, several studies in mice indicate that persistent parasites are important to maintain durable, anti-*Leishmania *memory responses [12,16]. These findings have led to the exploration of the use of live, genetically modified-parasites as an appealing strategy for developing vaccines against leishmaniasis [17,18]. Defined genetic alterations of the *Leishmania *genome can be achieved through homologous recombination [19], allowing disruption of essential genes for virulence and/or host survival. The first *Leishmania *mutant generated by gene replacement assayed as a potential *Leishmania *vaccine was an *L. major *line lacking the gene coding for dihydrofolate reductase-thymidylate synthase (DHFR-TS) [20]. This thymidine-auxotroph mutant was found to persist in BALB/c mice for up to 2 months, but it was incapable of causing disease. Interestingly, this *dhfr-ts *knockout was able to elicit substantial resistance in mice to a subsequent challenge with virulent *L. major*. However, immunizations with *dhfr-ts *knockouts derived from *L. chagasi*, *L. donovani*, or *L. major *did not protect against *L. chagasi *infection in BALB/c mice [21]. In another report, disruption of *BT1 *genes, encoding a biopterin transporter, in *Leishmania donovani *allowed the generation of a mutant line with reduced capacity for inducing infection in mice [22]. Furthermore, it was found that inoculation of BT1 null parasites elicits protective immunity in mice against an *L. donovani *challenge. Another mutant assayed as attenuated vaccine has been an *L. major *LPG2-knockout, which cannot synthesize LPG and other phosphoglycans; despite this defect, upon infection of mice, the mutants persist for several months without causing disease [23]. When BALB/c mice infected with *lpg2^- ^*parasites were challenged with virulent *L. major*, they were protected from disease [24]. However, the *in vivo *follow-up of these mutants led to the identification of a compensatory mutant (lpg2-REV) that regained virulence even in the absence of phosphoglycan synthesis [25]. In addition, it was found that *L. mexicana lpg2^- ^*mutants retained their virulence for macrophages and mice [26]. Another genetically-modified *L. mexicana *line, lacking cysteine proteinase genes *cpa *and *cpb*, was successfully used to protect against homologous infection in mice and hamsters [27,28]. Likewise, vaccination of BALB/c mice with an *L. mexicana *null mutant for GDP-mannose pyrophosphorylase (GDP-MP) conferred significant and long lasting protection against infection with virulent parasites [29]. Recently, it has been shown that BALB/c mouse infection with *L. infantum *mutant lacking one of the two *SIR2 *(silent information regulatory 2) alleles induced a high degree of protection against a virulent challenge [30]. More recently, it was reported that immunization with a *centrin *deletion mutant of *L. donovani *protected mice against infection with either *L. donovani *or *L. braziliensis *[31].

In this study, we analyzed the inmunoprotective ability of an *L. infantum *deletion mutant, lacking *HSP70 *type II gene (ΔHSP70-II), as a live vaccine against leishmaniasis in the *L. major*-BALB/c infection model. Immunization with this mutant line elicited specific immune responses and significant levels of protection against a challenge with virulent *L. major *promastigotes.

## Methods

### Animals and parasites

Experiments were performed in accordance with procedures approved by the Spanish Research Council Bioethics Committee. For animal experimentation, we followed the ethical principles dictated by the European Commission (Directive 86/609/CEE) for use of laboratory animals.

Female BALB/c and BALB SCID (CB-17*^scid^*) mice (6-8 week old), and male hamsters (*Mesocricetus auratus*; 8 week old) were purchased from Harlan Interfauna Iberica S.A. (Barcelona, Spain) and maintained in specific-pathogen-free facilities.

The ΔHSP70-II null mutant (*Δhsp70-II::NEO/Δhsp70-II::HYG*) is a cloned line that was generated by targeted deletion of both *HSP70-II *alleles in the *L. infantum *strain MCAN/ES/96/BCN150 [32]. *L. major *promastigotes (strain MHOM/IL/80/Friedlin; clon V1) were also used in this study. Promastigotes of both species were grown in RPMI 1640 culture medium supplemented with 10% heat-inactivated FBS, 100 U/ml penicillin and 100 μg/ml streptomycin.

### Infections

The virulence of *L. infantum *and *L. major *parasites was maintained by passage in hamsters and BALB/c mice, respectively. For infections, amastigote-derived promastigotes with less than 3 passages *in vitro *were used. For infection of mice with the ΔHSP70-II mutant (10^7 ^promastigotes/mouse), three routes were assayed: intravenous (IV; tail-vein injection), intraperitoneal (IP), and subcutaneous (SC; right hind-footpad). Mice from the control groups were inoculated with 0.1 ml of PBS.

Hamsters were infected with 5 × 10^7 ^ΔHSP70-II promastigotes by the intracardiac route (IC) as described elsewhere [33]. Age- and sex-matched hamsters were maintained uninfected and used as control for immunological determinations.

*L. major *metacyclic promastigotes were purified from stationary phase cultures. Briefly, promastigotes were resuspended in phosphate buffer saline (PBS) at 10^8 ^cells/ml, and peanut agglutinin (Vector laboratories) was added at 50 μg/ml; the sample was incubated for 25 min at room temperature. After centrifugation at 900 × g for 10 min, the supernatant contained the non-agglutinated metacyclic promastigotes. A thousand of metacyclic promastigotes in 50 μl were injected in the right hind-footpad of BALB/c mice. The growth of the lesion was monitored at indicated time points by measuring the thickness of the footpad using a dial caliper. The contralateral footpad of each animal represented the control value and the swelling calculated as: thickness of the right footpad - thickness of the left footpad.

Determination of the tissue parasite burden was carried out by quantitative limiting-dilution as described by Buffet and co-workers [34]. Briefly, whole lymph nodes, spleens and weighed pieces of liver were individually homogenized in Schneider's medium supplemented with 20% inactivated-FBS, 100 U/ml penicillin and 100 μg/ml streptomycin. The homogenates, in quadruplicate, were serially diluted across 96-well plates. Wells were examined for the presence of motile parasites after 2 (for *L. major*) or 4 (for *L. infantum*) weeks of culturing at 26°C. The number of parasites per organ was calculated as follows: parasite burden = (geometric mean of titer from quadruplicate cultures) × (reciprocal fraction of the homogenized organ inoculated into the first well). The titer was the reciprocal of the last dilution in which parasites were observed.

### Analysis of antibody responses

A *Leishmania *crude antigen was prepared from *L. infantum *promastigotes by incubation of microorganisms in lysis buffer (1% Triton X-100, 150 mM NaCl, 10 mM Tris-HCl pH 8 and 1 mM PMSF) for 15 min. Afterwards, the suspension, kept on ice, was sonicated until a decrease in viscosity was observed. The insoluble material was pelleted at 10 000 × g for 5 min and the supernatant was immediately stored at -70°C until use.

Sera were taken from all groups of mice before and 4 weeks after inoculation of the ΔHSP70-II promastigotes. Serum samples were analyzed for specific antibodies against *Leishmania *total antigen by standard ELISA assay. Briefly, standard plates (NUNC A/S, Roskilde, Denmark) were coated overnight at 4°C with 100 μl of *Leishmania *crude antigen (2 μg/ml in PBS). The sera from mice were assayed using two-fold serial dilutions. As secondary antibodies the following peroxidase-conjugates (Nordic Immunology Laboratories, Tilburg, The Netherlands) were used: goat anti-mouse IgG1 (1:1000 dilution) and goat anti-mouse IgG2a (1:1000 dilution). Orthophenylenediamine dihydrochloride (DAKO A/S, Glostrup, Denmark) was used as a peroxidase substrate. After 30 min, the reaction was stopped by the addition of 100 μl of 1 M H_2_SO_4_, and the absorbance was read at 450 nm. Titer was calculated as the reciprocal of the serum dilution that gave an absorbance above the mean value of preimmune sera plus three standard deviations.

Antibody responses in hamsters were determined by ELISA using the same antigen and coating conditions indicated above. The primary sera were assayed at 1:200 dilution, and the secondary antibody (RAHa/IgG(H+L)/PO conjugate; Nordic) was used at 1:1500 dilution.

### Nitric oxide (NO) determinations

Peritoneal macrophages from the different groups of mice (either control or infected with the ΔHSP70-II mutant by different routes, see above) were cultured at a concentration of 10^6 ^cells per milliliter in RPMI 1640 culture medium supplemented with 10% heat-inactivated FBS, 100 U/ml penicillin and 100 μg/ml streptomycin. Soluble *Leishmania *antigen (SLA; see below) was added to a final concentration of 50 μg/ml. After 48 h of incubation at 37°C, cell-free supernatants were collected and the nitrite level was stimulated using the Griess reaction kit (Sigma-Aldrich) according to the manufacturer's protocol. The basal NO production by murine macrophages was determined after culturing of the cells in the absence of SLA.

### Lymphoproliferation assays

At nine months after infection with ΔHSP70-II promastigotes, hamsters (n = 4) were euthanized and single cell suspensions from the spleens were made. Splenocytes from age- and sex-matched hamsters were used as controls. Cells (2.5 ×10^6^/ml) were plated in 96-well plates (200 μl) in RPMI medium supplemented with 10% heat-inactivated FBS, 100 U/ml penicillin and 100 μg/ml streptomycin. Cells were stimulated with either Concanavalin A (ConA; 1 μg/ml) or SLA (10 μg/ml) for 72 h. As control, unstimulated cultures were maintained in parallel. Proliferation was evaluated after addition of 1 μCi of [^3^H]thymidine (5 Ci/mmol) for the last 16-h of incubation. Incorporation of thymidine was assessed by scintillation counting.

SLA was prepared by three freezing and thawing cycles of stationary promastigotes of *L. infantum *suspended in PBS. After cell lysis, soluble antigens were separated from the insoluble fraction by centrifugation for 15 min at 12,000 ×g.

## Results and discussion

### Inoculation of ΔHSP70-II parasites protects BALB/c mice against *L. major *challenge

In a previous work [35], we found that *L. infantum *parasites lacking the *HSP70-II *gene (ΔHSP70-II) have a virulence greatly reduced. Thus, after infection of BALB/c mice with 10 millions of ΔHSP70-II promastigotes, the parasite loads in liver and spleen were more than 1000-fold lower than those achieved after infection with wildtype (WT) parasites. Nevertheless, the immune response elicited in mice by the infection with ΔHSP70-II parasites showed immunoprotective features. Thus, even though mice infected with ΔHSP70-II promastigotes had low levels of *Leishmania*-specific antibodies, the IgG2a/IgG1 ratio was higher in ΔHSP70-II infected mice than in mice infected with the WT promastigotes [35]. IgG2a dominance in the immune response is associated with protective responses against *Leishmania *infection [36].

**Figure 1:**
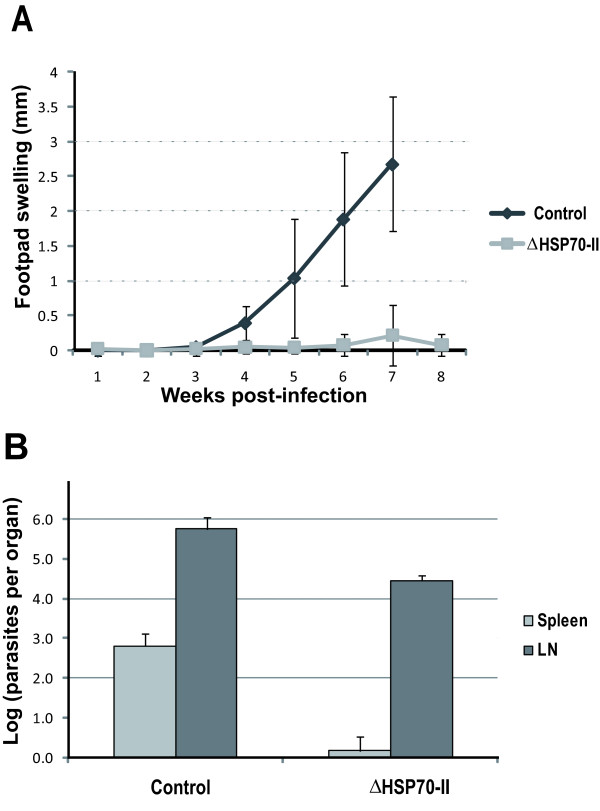
**Course of *L.major *infection in BALB/c mice vaccinated with ΔHSP70-II promastigotes**. BALB/c mice (n = 5) were inoculated with 1 × 10^7 ^ΔHSP70-II promastigotes and, 4 weeks later, challenged with 1,000 metacyclic promastigotes of *L. major*. The control group (unvaccinated) was challenged with an identical inoculum of *L. major*. (A) Course of lesion progression. (B) Parasite burdens in spleen and popliteal lymph node (right foot). The data presented are representative of two experiments with similar results.

To investigate whether ΔHSP70-II parasites would provide protection to leishmaniasis, we used the *L. major*-BALB/c infection model. In many ways, the infection of BALB/c mice with *L. major *may be a better model for human VL than for CL [37,38]. These mice develop high antibody titers, and the parasites frequently metastasize to distant locations including the bone marrow, liver, and spleen. Similar to human VL, BALB/c mice typically succumb to infection with *L. major *[39]. Nevertheless, it should be clearly stated that the hamster and the dog are better animal models than the mouse for human VL [40]. For the experiment, mice were intravenously infected with 10^7 ^ΔHSP70-II promastigotes, and 4 weeks later were challenged in the footpad with 1000 *L. major *metacyclics. Control mice developed large, nonhealing cutaneous lesions and had to be euthanized after 7 weeks. In contrast, mice previously infected with ΔHSP70-II parasites displayed a resistance phenotype: a reduced inflammation was observed in one mouse, and no lesions appeared in the rest of mice (Figure 1A). Mice were sacrificed and the parasite burden determined in the draining popliteal lymph node and spleen by limiting dilution (Figure 1B). In agreement with lesion progression, mice previously infected with ΔHSP70-II parasites had fewer parasites than controls; the differences were particularly dramatic when comparing the parasite loads in the spleen. Dissemination of *L. major *parasites to internal organs correlates with susceptibility in the mouse infection model [38]. Thus, inoculation of *L. major *promastigotes into the dermis or the footpad of BALB/c mice leads to the dissemination of the parasite, after a short period (10-24 hours), to the spleen, the liver, the bone marrow, and, occasionally, the kidney. However, in similarly infected mouse strains with a curative phenotype like C57BL/6, CBA/J, and C3H/HeJ, the parasites remain localized in the footpad and in the draining popliteal lymph node for many days without evidence of dissemination. In conclusion, the results indicated that an effective protection was attained in BALB/c mice infected with the ΔHSP70-II mutant.

### Immunological and parasitological parameters associated with the inoculation route

**Figure 2:**
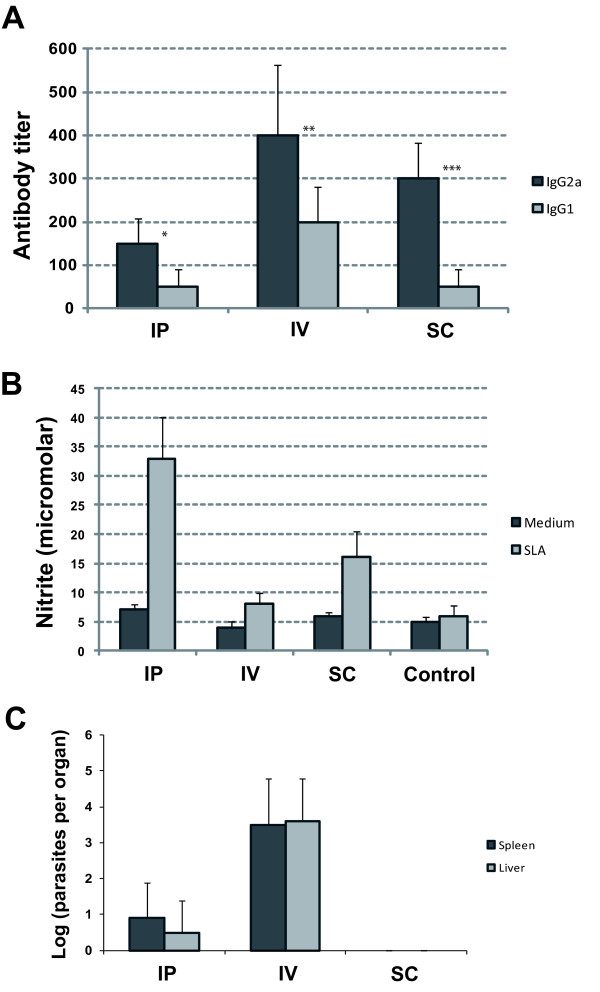
**Immunological and parasitological parameters associated with the inoculation route of ΔHSP70-II promastigotes in BALB/c mice**. Groups of mice (n = 4) were inoculated with 1 × 10^7 ^ΔHSP70-II promastigotes by the intraperitoneal (IP), intravenous (IV) or subcutaneous (SC) route. (A) Four weeks after infection the anti-*Leishmania *specific IgG1 and IgG2a titers were determined by ELISA. Differences between IgG1 and IgG2a titers for each group of mice were compared by using the two-tailed paired Student's *t*-test: *, p = 0.016; **, p = 0.066; ***, p = 0.019. (B) Production of NO by peritoneal macrophages from ΔHSP70-II infected (IP, IV, and SC) or control mice. (C) Parasite burdens in spleen and liver of ΔHSP70-II infected mice. The data are represented as the mean + standard deviation.

The above experiments demonstrate that IV inoculation with ΔHSP70-II parasites confers a significant protection against *L. major *infection in mice. In a new set of experiments, we compared the immunological responses observed after inoculation of the mutant by three inoculation routes: intraperitoneal (IP), IV and subcutaneous (SC). We first focused on the humoral immune response and determined the titers of IgG1 and IgG2a antibodies against *Leishmania *proteins by ELISA before and 4 weeks after inoculation of the mutant line in all three groups. As shown in Figure 2A, the IgG2a titers were higher than IgG1 titers for all groups, suggesting that infection with ΔHSP70-II parasites, independent of the inoculation route, leads to a predominant production of anti-*Leishmania *antibodies of the IgG2a isotype. Although inoculation through the IV route leads to considerably higher titers (2-4 fold) of IgG2a antibodies in comparison to the other inoculation routes (Figure 2A), the highest IgG2a/IgG1 was observed in mice infected through the SC route. IgG2a antibody formation is dependent on IFN-γ as an IgM-to-IgG2a switch factor and is considered to be typical for a T helper type 1 (Th1) response; in contrast, IgG1 production depends on IL-4 secreted by Th2 cells [41].

Because the production of NO by macrophages is a key factor in killing *Leishmania*, we determined also the level of NO produced by peritoneal macrophages from ΔHSP70-II infected mice after *in vitro *re-stimulation with SLA. Interestingly, significant levels of nitrites were observed in the culture supernatants of macrophages of the three infection-groups when compared with control macrophages (Figure 2B). Nevertheless, production of NO was clearly higher by SLA-stimulated macrophages from mice infected with ΔHSP70-II parasites through the IP route.

Finally, we analyzed the parasite burden in spleen and liver of the different groups of ΔHSP70-II infected mice (Figure 2C). No parasites were detected in BALB/c mice four weeks after infection through the SC route suggesting that the ΔHSP70-II mutant was unable to visceralize. On the other hand, the parasite burden was higher when the mutant is inoculated through the IV route than when the mutant is inoculated into the peritoneal cavity.

### ΔHSP70-II parasites are highly attenuated in susceptible *L. infantum *animal models

Before considering the ΔHSP70-II mutant line as a candidate vaccine, it is critical that the parasites remains attenuated, and that a selection of escape variants, in which the infectivity is restored, does not occur as a consequence of continuous passage in BALB/c mice. Spontaneous recovery of virulence has been observed for some attenuated *Leishmania *mutants, e.g. lpg2^- ^[25], LmxPK4^- ^[42], and hsp100^- ^[43].

**Figure 3:**
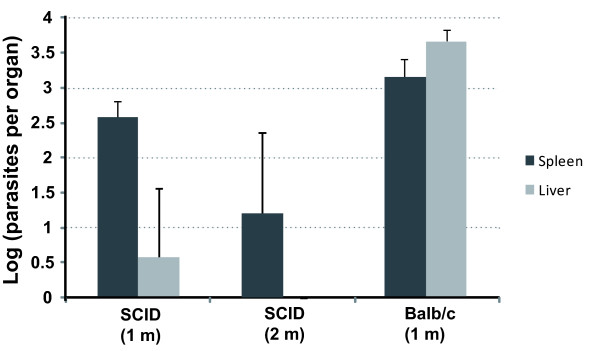
**Infectivity of ΔHSP70-II promastigotes in immunocompromised SCID mice**. Groups (n = 4) of SCID mice were inoculated with 1 × 10^7 ^ΔHSP70-II promastigotes and the parasite burdens were determined in spleen and liver after 1 or 2 months post-infection. As control, a group (n = 4) of BALB/c was similarly infected, and the parasite burdens were determined 1 month post-infection.

The ΔHSP70-II mutant line was created in 2005 [32]; since then, the parasite has been used many times to infect BALB/c, and recovery of virulence has not been observed (see also Figures 2 and 3). To further explore whether the ΔHSP70-II parasites would remain attenuated in the absence of a functional immune system, SCID mice lacking functional T and B cells were infected. As shown in Figure 3, SCID mice infected with ΔHSP70-II promastigotes showed even lower parasite burdens than ΔHSP70-II-infected BALB/c mice (this is especially true for the liver). At first glance this result may be considered unexpected; however, after carefully revising previous studies on *Leishmania *infection of SCID mice, our findings may be expected. Certainly, the parasite burdens in SCID mice are higher than those observed in immunocompetent mice, but it is true at long-term. However, if the parasite burdens are determined at short-term, the parasitemia is lower in the SCID mice than in the BALB/c [30,44]. The reason may be in the fact that SCID mice, perhaps as a compensatory mechanism due to the lack of functional T and B cells, have a relatively higher potential of functional NK cells (see Solbach and Laskay [39] for further details). As the ΔHSP70-II line has an intrinsic low capacity of multiplication in mammalian hosts, the higher activity or levels of NK cells in SCID may explain why the parasite loads are lower in SCID mice than in BALB/c mice.

On the other hand, in the initial experiments of BALB/c infection with this mutant line, no parasites were recovered in the liver [35]; thus, it is likely that the ΔHSP70-II parasite has evolved a liver tropism after *in vivo *passing. Nevertheless, it should always be kept in mind that the parasite burdens in ΔHSP70-II infected BALB/c mice were three to four orders of magnitude lower than those found in mice infected with the *L. infantum *parental strain (BCN150); as reported previously [35], at 4 weeks post-infection, the total burdens per organ, in BCN150-infected BALB/c mice, were around 2 million (6.3 logarithmic units) in spleen, and 80 million (7.9 logarithmic units) in liver. Even more interestingly, we found that parasite burdens in ΔHSP70-II-infected SCID declined at two months post-infection and parasites were not detected in the liver at this time (Figure 3). This finding demonstrates that ΔHSP70-II parasites cannot replicate efficiently even in immunocompromised SCID mice, which otherwise when infected with *L. infantum *WT parasites develop a progressive parasitemia [30]. Together, those results confirm that ΔHSP70-II parasites would constitute a safe vaccine.

Inbred strains of mice are not adequate models for infectivity analysis of viscerotropic strains of *Leishmania*, as mice naturally develop protective immunity against the infection that leads to a clearance of the parasite [45]. Thus, we further assessed the virulence of the ΔHSP70-II mutant in the golden hamster (*M. auratus*) model, which is a laboratory animal that accurately reproduce pathological aspects of human VL, such as an uncontrolled parasite replication in the liver and spleen [33,46]. Furthermore, golden hamsters are so susceptible for *L. infantum *infection that infective doses as low as 1000 parasites result in fatal disease [33].

A group of four hamsters were intracardially inoculated with 50 million ΔHSP70-II promastigotes and the animals were examined weekly for clinical symptoms of disease progression. Additionally, at two-month intervals, blood samples were obtained and the humoral response against *Leishmania *was assayed. Although weak, a specific anti-SLA antibody response was detected at two months post-infection in all animals (data not shown). Afterwards, the IgG reactivity decreased slightly, but a specific response still remained detectable at 9 months post-infection (Table 1). Apart from this, no other signs of infection were observed in the ΔHSP70-II-infected hamsters; instead the animals remained healthy. At this point, 9 months post-infection, animals were sacrificed and individually analyzed for the present of parasites in liver and spleen by limiting dilution. No parasites were found in any of the tissues, and the organs showed a normal morphology. In parallel, the capacity of spleen cells, from infected and control hamsters, to proliferate in the presence of parasite antigens was assessed (Table 1). Remarkably, splenocytes from ΔHSP70-II-infected hamsters specifically proliferated in response to *Leishmania *antigens (2-3 fold above control cells). Furthermore, splenocytes of ΔHSP70-II-infected hamsters showed a proliferation capacity, similar to control animals, after stimulating with the mitogen ConA (Table 1). It should be noticed that infection of hamsters with virulent *L. donovani *(and also with *L. infantum*; our unpublished data) leads to a significant suppression of the ability of spleen cells to respond to ConA [47]. In summary, these data suggest that inoculation with the ΔHSP70-II mutant was able to elicit a long-lasting immune response, without affecting the normal function of the immune system.

**Table 1:** Immunological responses elicited in golden hamsters infected with ΔHSP70-II parasites

		**Proliferation ± SD (cpm)**^**a**^		
	**IgG reactivity^a^**	**Medium**	**SLA**	**ConA**

Uninfected	0.12 ± 0.01	4444 ± 840	4467 ± 674	230427 ± 14932
ΔHSP70-II	0.25 ± 0.03	4646 ± 763	9515 ± 1953	264887 ± 12337

^a^See Methods for experimental details

## Conclusions

A vaccine against leishmaniasis seems to be feasible since most individuals that were once infected with *Leishmania *become resistant to clinical infection when later exposed to the parasite. However, despite great research effort, leishmanization with live *Leishmania *parasites remains the only vaccine with proven efficacy against human leishmaniasis. Genetic modification of *Leishmania *to reduce virulence, yet maintaining immunogenicity, is of current interest in vaccine research. According to the levels of IgG1 and IgG2a antibodies, and the NO production, the immunization of mice with the ΔHSP70-II deletion mutant appears to be eliciting predominantly Th1 responses, independently of the route of administration (intraperitoneal, intravenous or subcutaneous). In addition, we found that immunization of BALB/c with ΔHSP70-II promastigotes lead to an effective immune response able to protect these mice against infection with *L. major*.

In summary, present results offer hope for the development of a live-attenuated vaccine against *Leishmania *based on this mutant line. However, we are aware that this work constitutes a preliminary study and that further experiments, using more appropriate models for LV (hamsters and/or dogs), are needed. An important concern with live-attenuated vaccines is their safety, as there are fears that the parasite may revert back to a virulent form or cause lesions in immunesuppressed individuals. Interestingly, the low numbers of parasites found after infection with ΔHSP70-II promastigotes in hamsters, BALB/c mice and even in SCID mice (lacking both T and B cells) support the idea that this mutant would be a safe vaccine, which might be helpful to design prevention strategies against *Leishmania *infection in both dogs and humans [48]. Additionally, based on this safety, this Δhsp70-II line could also have usefulness as a platform for introduction of immunoprotective antigens relevant to leishmaniasis or even to other diseases.

## Competing interests

The authors declare that they have no competing interests.

## Authors' contributions

JC carried out most of the experimental procedures. CF constructed the ΔHSP70-II mutant line. MS and JMR performed immunoproliferation assays. MF and JMR conceived the research, contributed with data analysis and revision of the manuscript. JMR wrote the manuscript. All authors read and approved the final version of the manuscript.
